# Antioxidant Content of Frozen, Convective Air-Dried, Freeze-Dried, and Swell-Dried Chokecherries (*Prunus virginiana* L.)

**DOI:** 10.3390/molecules25051190

**Published:** 2020-03-06

**Authors:** Carmen Téllez-Pérez, Anaberta Cardador-Martínez, Viridiana Tejada-Ortigoza, Marla C. Soria-Mejía, Iván Balderas-León, Maritza Alonzo-Macías

**Affiliations:** Departamento de Bioingenierias, Tecnologico de Monterrey, Monterrey 76158, Mexico; ctellezperez@gmail.com (C.T.-P.); mcardador@tec.mx (A.C.-M.); viri.tejada@tec.mx (V.T.-O.); marlasoriamejia@gmail.com (M.C.S.-M.); balderasleon90@gmail.com (I.B.-L.)

**Keywords:** chokecherry, *Prunus virginiana* L., freezing, drying, phenolics, antioxidant activity, swell drying

## Abstract

Chokecherry (*Prunus virginiana* L.) is rich in bioactive molecules as phenolics, which can act as antioxidants, anti-inflammatory, anticancer, among others; however, due to its high perishability, most of this fruit is wasted. Freezing and sun drying have been the most adopted techniques to avoid its postharvest deterioration. Nevertheless, both processes have presented some drawbacks as high storage costs and losses of bioactive molecules. Therefore, to preserve these molecules, this study compared the impact of convective airflow drying (CAD), freezing (FR), freeze drying (FD), and swell drying (SD). Total phenolics content (TPC), total flavonoids content (TFC), kuromanin concentration (KC), and antioxidant activity (antiradical activity (ARA) and Trolox equivalent antioxidant capacity assay (TEAC)) of chokecherries were measured. “Swell drying” is a drying process coupling convective airflow drying to the Instant Controlled Pressure Drop (DIC) expansion. A central composite rotatable design was applied to optimize the DIC variables and responses. Results showed that both freezing and swell drying effectively preserve the TPC, TFC, KC, and ARA. Moreover, SD samples also presented the highest TEAC. Contrary, in the case of CAD, it caused the highest losses of both antioxidant content and activity. Swell drying remedies the shrinkage and collapsing of dried food structure, which results in a better antioxidants extraction.

## 1. Introduction

Consumer awareness about the health implications of ingesting fruit and vegetables has increased recently. Fruit phenolic compounds have been studied due to their antioxidant potential and important consequences in health maintenance, such as potential prevention and protection from diseases such as cancer or cardiovascular disease [1].

Among fruit, wild cherries have recently attracted attention due to its high antioxidant activity resulted from its pigment compounds, such as anthocyanins and procyanidins [2,3]. Chokecherry (*Prunus virginiana* L.) is a wild cherry from a shrub of the *Rosaceae* family native of North America. Its common name “chokecherry” comes from the astringency of the fruit caused by its antioxidant compounds [4]. Chokecherries are dark red to purple small spherical fruit (6 to 8 mm in diameter) with a single stone [5]. They have been widely used as food and for medicinal purposes by Native Americans in its raw and home processed form (juice, jellies, wine, syrups, dried, etc.) [4,6,7,8]. 

Recent studies have shown that chokecherries are rich in anthocyanins as cyanidin 3-glucoside and cyanidin 3-rutinoside [2]. Anthocyanins are one of the major groups of pigments belonging to the secondary metabolite group of flavonoids [9]. However, long thermal treatments of traditional food processing cause losses in anthocyanin content (~50%) and formation of browning compounds [10].

Traditionally, chokecherries have been dried on grass mats in the sun and then stored [4,8]. Nevertheless, because of its high moisture content (~84% w.b.), chokecherry is exposed to several reactions that led to its spoilage and loss of quality and nutrimental content.

Convective airflow drying (CAD) has also been used to preserve fruit. Nevertheless, extended periods and high temperatures lead to undesirable changes (shrinkage and collapsing), and the degradation of main phytochemicals such as antioxidant compounds [11].

Freezing (FR) allows the preservation of food attributes such as taste and volume; however, this process is considered expensive mainly due to the high initial investment cost for the equipment and the storage cost of frozen fruit [12]. Moreover, when cold storage temperatures fluctuate above −18 °C thawing takes place, which could trigger nutrient losses.

Freeze drying (FD) is an excellent alternative to preserve labile and photo-oxidative compounds in food as antioxidants; however, as compared to convective air drying, FD costs are four to eight times higher [13]. This cost includes special packaging, refrigeration, freezing and vacuum equipment, and high energy consumption during the whole drying process [14].

In response, novel emerging technologies have been applied to improve the preservation of natural food compounds. “Swell drying” is a unique drying process coupling convective airflow drying to the instant controlled pressure drop (DIC) expansion. This process is well known by guaranteeing (1) the preservation of functional properties, (2) the organoleptic quality, (3) the effective microbiological and fungi decontamination, and (4) the reduction of energy consumption and the drying time. DIC by its French acronym “*Détente Instantanée Contrôlée*,” is a high temperature/short time (HTST) process that improves both performance of the drying process and high-quality functional foods [15]. This technology consists of subjecting the food material to high-pressure and high-temperature treatments (from 0.1 up to 1 MPa) followed with an abrupt pressure drop (ΔP/Δt > 0.5 MPa s^−1^) towards a vacuum (around 4.5 kPa). After pressure drops, several thermo-mechanical effects occur in the material, such as the rapid vaporization of volatile compounds, expansion, and instantaneous cooling of the products [11]. Moreover, thanks to the newly generated porous structure by DIC, the final stage of drying can be reached more quickly. Thus, swell drying reduces energy consumption and, consequently, manufacturing costs as compared with convection air drying.

Therefore, this work aimed to evaluate the effect of convective airflow drying (CAD), freezing (FR), freeze drying (FD), and swell drying (SD) on the antioxidant content and the antioxidant activity of chokecherry fruit (*Prunus virginiana* L.). A central composite rotatable design was applied to evaluate the effect of the instant controlled pressure drop technology operating parameters (steam pressure and thermal treatment time) on variables responses of swell-dried chokecherries.

## 2. Results

### 2.1. Proximal Analysis

Proximate analysis of dried chokecherries (g/100 g of dry solids) presented crude protein (nitrogen × 6.25) of 0.38 ± 0.00 g, total lipids of 1.54 ± 0.28 g, ash of 10.83 ± 0.67 g, crude fiber of 1.47 ± 0.27, and total carbohydrates (calculated by difference) of 85.78 ± 0.39 g. The moisture contents of frozen and dried chokecherries are shown in Table 1.

### 2.2. Bioactive Compounds Quantification

Total phenol content (TPC), total flavonoids content (TFC), and kuromanin concentration (KC) varied widely according to different drying and freezing conditions applied to chokecherries. Table 1 and Table 2 show the obtained results for these bioactive compounds.

### 2.3. Total Phenolics Content

**Figure 1:**
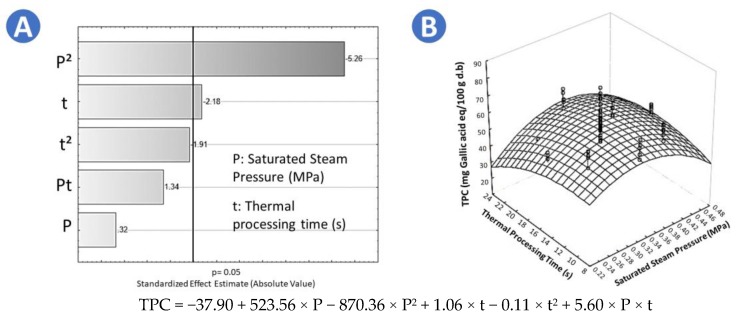
Effects of steam pressure (MPa) and thermal processing time (s) on the total phenol content (TPC) of swell-dried chokecherries. (**A**) Pareto chart and (**B**) surface response plot.

As shown on Table 1, FR samples presented the highest TPC (72.81 mg gallic acid eq./100 g d.b.), followed by SD1 (65.57 mg gallic acid eq./ 100 g d.b.), FD (51.32 mg gallic acid eq./ 100 g d.b.), and CAD (50.29 mg gallic acid eq./100 g d.b.). Moreover, the Tukey test indicated that there was not a significant difference in the TPC between FD and CAD. On the other hand, regarding the Pareto chart in Figure 1A, it could be observed that in the case of swell-dried samples, the thermal holding time (t) and the quadratic effect of the saturated steam pressure (P^2^) had a significant effect on increasing the TPC. Therefore, the higher the treatment time, the higher the TPC of SD samples. In addition, as shown in Figure 1B, it could be highlighted that the optimal DIC treatment conditions to maximize the TPC were *p* = 0.35 MPa and t = 16 s. Adequate empirical regression model of the TPC of swell-dried chokecherries is shown in Figure 1.

### 2.4. Total Flavonoids Content

**Figure 2:**
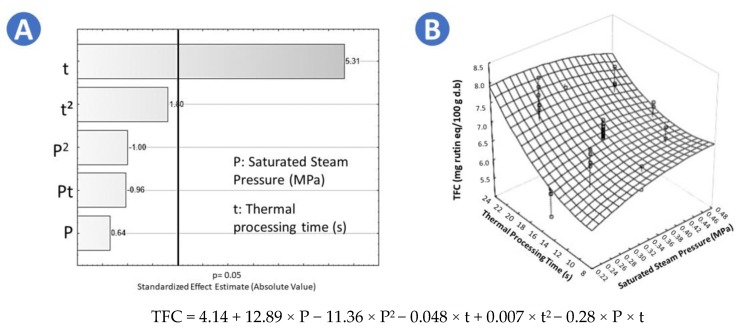
Effects of steam pressure (MPa) and thermal processing time (s) on the total flavonoids content (TFC) of swell-dried chokecherries. (**A**) Pareto chart and (**B**) surface response plot.

In the case of TFC, the highest value was performed by SD9 (*p* = 0.27 MPa and t = 20 s) with 7.81 mg rutin eq./100 g d.b. With respect to FR and FD samples, according to Tukey test they were not significantly differenent (7.02 mg rutin eq./100 g d.b. and 7.19 mg rutin eq./100 g d.b., respectively Table 1). The lowest TFC values were performed by SD11 (*p* = 0.24 MPa and t = 16 s) and CAD (5.88 mg rutin eq./100 g d.b. and 6.10 mg rutin eq./100 g d.b., respectively). With respect to the Pareto chart in Figure 2, it is shown that in the case of swell-dried samples, the thermal holding time (t) has a significant effect on increasing the TFC. By applying the surface response methodology to data, it was found that the optimal treatment conditions to maximize the TFC were *p* = 0.30 MPa and t = 22 s. Adequate regression model of the TFC of swell-dried chokecherries is shown in Figure 2.

### 2.5. Kuromanin Concentration

**Figure 3:**
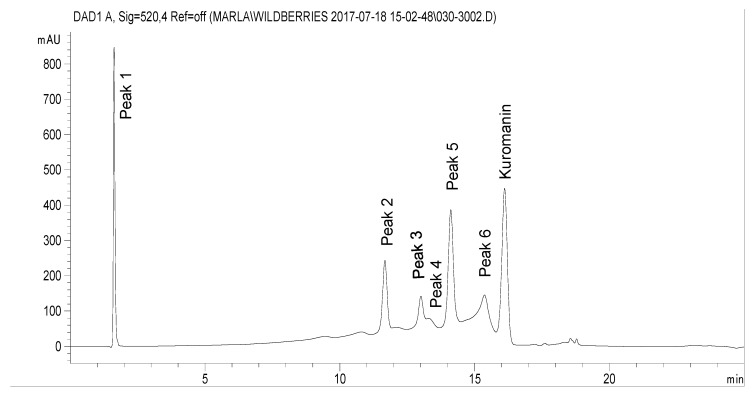
HPLC chromatogram of freeze-dried chokecherry. Anthocyanins are identified as Peak 1 to 6 and kuromanin according to the retention time of a standard.

**Figure 4:**
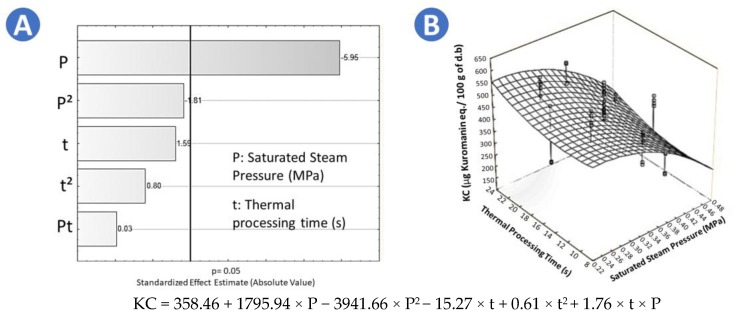
Effects of steam pressure (MPa) and thermal processing time (s) on the kuromanin concentration (KC) of swell-dried chokecherries. (**A**) Pareto chart and (**B**) surface response plot.

Table 2 shows the results of the chromatographic quantification of anthocyanins present in chokecherry after several treatments. The anthocyanins were labeled according to retention time as Peak 1 to Peak 6, the last peak was identified as kuromanin (a standard of kuromanin was used for this purpose, Figure 3). The concentration of anthocyanins 1 to 6 decreases their concentration in all swell drying treatments, probably due to temperature sensitivity. Because kuromanin was the most abundant anthocyanin, results are centered on its variation due to treatment. With respect to kuromanin concentration (KC), Table 2 shows that frozen samples presented the highest KC (758.06 µg kuromanin eq./100 g of d.b.). Moreover, by comparing drying operations, it is demonstrated that FD (640.24 µg kuromanin eq./100 g of d.b.) and SD9 (558.39 µg kuromanin eq./100 g of d.b.) allowed to preserve KC much better than CAD (107.99 µg kuromanin eq./100 g of d.b.). In the case of swell drying treatment, the Pareto chart shows that saturated steam pressure (P) has a significant effect on the preservation of kuromanin (Figure 4A). The higher the saturated steam pressure, the lower the KC of SD samples. By applying the surface response methodology to data (Figure 4B), it was found that the optimal treatment conditions to maximize the KC were *p* = 0.24 MPa and t = 22 s. Adequate regression model of the KC of swell-dried chokecherries is shown in Figure 4.

### 2.6. Antioxidant Activity

Results of the antiradical activity by DPPH (%ARA) and the Trolox equivalent antioxidant capacity (TEAC) assay of frozen and dried samples are shown in Table 3.

### 2.7. Antiradical Activity by DPPH (ARA)

**Figure 5:**
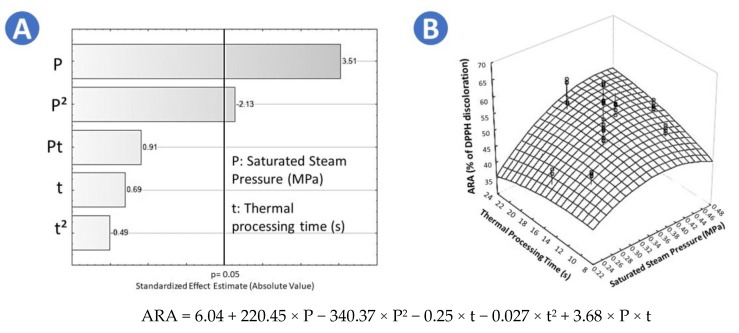
Effects of steam pressure (MPa) and thermal processing time (s) on the antiradical activity by DPPH of swell-dried chokecherries. (**A**) Pareto chart and (**B**) surface response plot.

FR and SD1 samples presented the highest ARA (61.38% and 62.89%, respectively, Table 3); furthermore, according to the Tukey test, there was not a significant difference between them. However, by comparing these results to CAD samples (33.97%) and FD samples (49.18%), it is highlighted that CAD halved the ARA of chokecherries, and FD reduced the ARA by 21%. In the case of the swell-dried samples, the Pareto chart showed that saturated steam pressure (P) and the quadratic effect of the saturated steam pressure (P^2^) had a significant effect on increasing the ARA of chokecherries (Figure 5A). The higher the saturated steam pressure, the higher the ARA of SD samples. Moreover, as shown in the surface response plot of ARA of SD chokecherries (Figure 5B), the optimal treatment conditions to maximize the ARA were *p* = 0.44 MPa and t = 22 s. Adequate regression model of ARA of swell-dried chokecherries is shown in Figure 5.

### 2.8. Trolox Equivalent Antioxidant Capacity (TEAC)

**Figure 6:**
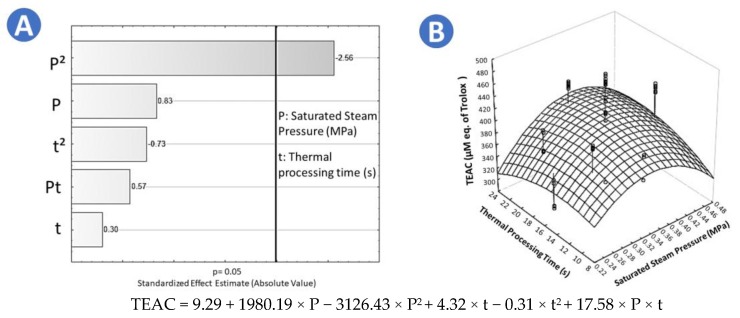
Effects of steam pressure (MPa) and thermal processing time (s) on the Trolox equivalent antioxidant capacity (TEAC) of swell-dried chokecherries. (**A**) Pareto chart and (**B**) surface response plot.

In the case of TEAC, the sample that presented the highest TEAC value was SD 1 (472.88), meaning that the antioxidant capacity of it was almost the same as the Trolox solution at the same concentration. Contrary, in the case of CAD sample, it was observed that TEAC was significantly reduced (111.30, Table 3). For the FR sample (436.26) and FD sample (450.55), results showed a slight reduction in TEAC by comparing them to SD 1. According to the Pareto chart (Figure 6A), for swell-dried samples, the quadratic effect of saturated steam pressure (P^2^) had a significant impact on the TEAC. As shown in Figure 6B, the optimal treatment conditions to maximize the TEAC of swell-dried chokecherries were *p* = 0.36 MPa and t = 17 s. Adequate regression model of TEAC of swell-dried chokecherries is shown in Figure 6.

## 3. Discussion

### 3.1. Proximal Analysis

By comparing our results to the study of Green and Low [5], it could be observed that our frozen samples presented a higher moisture content than fresh Canadian chokecherries (83.34% w.b. vs. 66.80% w.b.). The same tendency was also found on fat content (1.54 vs. 0.6 g/100 g of dry solids), ash content (10.83 vs. 2.71 g/100 g of dry solids), and total carbohydrate content (85.78 vs. 63.25 g/100 g of dry solids). Contrary, the protein content (6.62 vs. 0.38 g/100 g of dry solids) was higher on Canadian chokecherries. The variability on the chemical composition between both chokecherry samples could depend on various factors as the cultivar and variety, the plant nutrition, the ripeness stage at harvesting, as well as the growing location and the environmental conditions.

### 3.2. Bioactive Compounds Quantification

Phenolic compounds are secondary plant metabolites characterized by their in vitro and in vivo antioxidant activity, associated with their tendency to donate an electron or a hydrogen atom to a free radical [16]. The obtained results regarding the impact of freezing and drying on the TPC of chokecherries showed that freezing allowed a better extraction of these molecules (72.81 mg gallic acid eq./g d.b.). These results could be linked to the fact that freezing triggers the crystallization of water in the form of ice crystals; therefore, the size and location of the ice crystals could break cell membranes of fruits, and thereby release the phenolic compounds [17].

In the case of the impact of the studied drying methods (CAD, FD, and SD) on the TPC, the loss of ~31% of TPC of CAD samples with respect to frozen samples could be due extended exposure to oxygen and thermal deterioration of the phenolics during drying (24 h). Similar results were found by the study of Samoticha, et al. [18], who showed that convective drying at 50 °C reduced the TPC of fresh chokeberry samples ~38%. The loss of TPC of FD chokecherries (~29%) could be linked to a long desorption stage which could trigger a phenolic compounds damage. Regarding the TPC of swell-dried samples, it could be observed that according to DIC treatment conditions (saturated steam pressure and thermal treatment time), the TPC of SD chokecherries varied from 65.57 to 43.53 mg gallic acid eq./100 g d.b. Swell drying allowed the expansion of the predried fruit structure, which depended on the stress caused by the quantity of autovaporized water, the hydro-thermo-rheological behavior of the product, and the difference between the internal and external pressures [11]. Therefore, by using surface response methodology, it was possible to optimize the DIC treatment conditions to increase the availability of TPC of SD chokecherries (*p* = 0.35 MPa and t = 16 s).

Flavonoids are especially important antioxidants due to their high redox and chelating potential [19]. The obtained results showed that among all the freezing and drying operations, swell drying (*p* = 0.27 MPa and t = 20 s) preserved the best concentration of these compounds on chokecherries (7.81 mg rutin eq./100 g d.b.). Mounir [20] found similar results on apple and onion snacks where DIC texturing improved the flavonoid extraction significantly up to eight times more as compared with CAD. Freeze drying also effectively preserved the TFC (7.19 mg rutin eq./100 g d.b.) of chokecherries. Similar results have been found on strawberries and green Poblano pepper where both swell-dried fruit showed the same performance as FD to preserve flavonoids [21,22]. Loss of TFC on frozen chokecherries, could be caused by drip loss during thawing and to the presence of activated enzymes that degrade flavonoids. Loss on TFC of CAD chokecherries could be linked to shrinkage and collapsing of the fruit structure during drying, and because of flavonoids are thermolabile compounds, long convective hot air drying could provoke flavonoids losses.

Anthocyanins in plants are water-soluble vacuolar pigments (red, purple, or blue) that can act as antioxidants by donating hydrogen to highly reactive radicals [23]. To evaluate the effect of freezing and drying on chokecherries anthocyanins, the kuromanin (cyanidin 3-O-β-glucopyranoside) concentration was evaluated. Our results showed that as in the case of TPC, freezing effectively preserved the kuromanin content (758 µg kuromanin eq./100 g of d.b.). This result could be attributed to microstructural modifications due to freezing, such as cytoplasm disruption, that allowed a better kuromanin extraction. Nevertheless, freeze drying is the only drying method that allows food materials to dry without any noticeable shrinkage or collapse, the good extraction of kuromanin (640 µg kuromanin eq./100 g of d.b.) could be linked to a porous structure of FD chokecherries. With respect to swell drying, high saturated steam processing provoked kuromanin losses between 26.3% to 65.6% with respect to FR samples. These results could be associated with kuromanin structural sensitivity to temperature, light, and air [24]. Therefore, to optimize the kuromanin extraction of chokecherries by swell drying, it would be necessary to study a wide range of parameters as predrying conditions (temperature and airflow), predrying moisture content, low ranges of saturated steam pressure and thermal treatment time, postdrying conditions, and glass transition temperature being the main factors that influence the structure modifications; and thus the extraction process itself. Moreover, the study of DIC condensates could become a tool to evaluate the possible leach of kuromanin on the vacuum tank. Skrovankova, Sumczynski, Mlcek, Jurikova and Sochor [16] reported that in canned products, between 21% and 33% of berries’ anthocyanins were leached out into the brine. Finally, the important kuromanin losses of CAD samples respect to FR samples (~86%) could be linked to several factors as long drying time, light, oxygen, the presence of enzymes, among others [23]. Ou, Bosak, Brickner, Iezzoni and Seymour [24] found a similar behavior for the total anthocyanin content of frozen and dried cherries (*Prunus cerasus*); in their study, dried samples presented ~70% less anthocyanins than frozen samples. Giovanelli, et al. [25] also stated that air drying at 70 °C decreased by about 30% of the anthocyanin content of blueberries (*Vaccinium corymbosum*). According to Šumić, et al. [26], while drying temperature increases, anthocyanin content decreases. Chokecherries have high anthocyanin contents, however, because their stability is affected by several factors such as pH, temperature, light, etc. Its valorization has been limited, thus, it is important to optimize processing parameters to increase their bioavailability.

### 3.3. Antioxidant Activity

In the case of the impact of the studied preserving methods (CAD, FR, FD, and SD) on the antioxidant activity, it could be observed that for both the antiradical activity by DDPH (ARA) and the Trolox equivalent antioxidant capacity (TEAC), swell drying sample 1 (P = 0.35 MPa and t = 16 s) presented the highest antioxidant activity. Similar results were also found by Alonzo-Macías, Cardador-Martínez, Mounir, Montejano-Gaitán and Allaf [21], who studied the impact of swell drying on the antioxidant activity of strawberries (*Fragaria var. Camarosa*). Their results showed that swell drying increased the ARA of CAD and FD by 9% and 10%, respectively. Moreover, their optimum treatment conditions of DIC to obtain the highest levels of antioxidant activity (ARA) were 0.35 MPa and 10 s. The enhancement of the antioxidant activity of swell-dried samples could be linked to the fact that DIC leads to significant changes in the texture and structure of SD samples, which results in better antioxidants extraction.

Moreover, during the HTST DIC treatment, it could be allowing the formation of compounds with antioxidant activity such as Maillard reaction products. In fact, according to Turkmen, et al. [27] moderate heat treatment could be considered a useful tool for improving the antioxidant activity of some vegetables (pepper, broccoli, spinach, and green beans).

However, the reduction of ARA and TEAC of CAD samples could possibly be linked to the negative effect on the antioxidant molecules due to the long exposure time to high temperatures. Moreover, the compact structure of CAD samples did not allow adequate antioxidant extraction. With respect to the impact of freezing on the antioxidant activity, we concluded that under the studied conditions, the ARA and TEAC of chokecherries were well preserved. The formation of ice crystals during chokecherries freezing can damage the food microstructure, leading to a better antioxidant extraction. Finally, regarding the antioxidant activity of freeze-dried chokecherries, we observed that freeze drying exhibited effective preservation of antioxidant properties. Hence, the slight losses on the antioxidant activity of FD chokecherries could be linked to the long evaporative secondary drying.

## 4. Materials and Methods

### 4.1. Materials

#### 4.1.1. Biological Material

**Figure 7:**
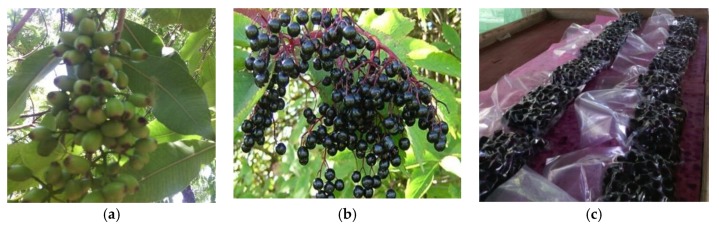
(**a**) A close up of immature chokecherries hanging from a tree; (**b**) a close up of mature chokecherries hanging from a tree; (**c**) frozen packaged chokecherries in vacuum bags.

Frozen chokecherries (*Prunus virginiana* L.) were obtained from FIAVHI (Fideicomiso de Infraestructura Ambiental de Los Valles de Hidalgo) Mexico. After harvesting, chokecherries were washed, sorted by maturation stages (Figure 7a,b), and manually pitted. The ratio between flesh and stone was around 3:1. Only ripe fruit was frozen and stored for less than a week until further experiments. Chokecherry fruit was considered mature when it was fully dark purple. Freezing was carried out at −20 °C in a horizontal freezer in high-density polyethylene vacuum bags (Figure 7c).

#### 4.1.2. Reagents and Solvents

Folin–Ciocalteu reagent, 2,2-diphenyl-1-picrylhydrazyl (DPPH), 2,2′-azino-bis (3-ethylbenzothiazoline)-6-sulfonic acid (ABTS), gallic acid, 1,6-hydroxy-2,5,7,8-tetramethylchroman-2-carboxylic acid (Trolox), potassium persulfate, and kuromanin standard were purchased from Sigma-Aldrich Canada Ltd., Oakville, ON, Canada. HPLC grade methanol was obtained from Karal (León; Guanajuato, Mexico) and used water was obtained from a Millipore Milli-Q water system with a resistivity of 18.2 MΩ cm (25 °C).

### 4.2. Methods

#### 4.2.1. Drying Procedures

Before any drying treatment, frozen chokecherries were divided into four batches as follows: freezing (FR), freeze drying (FD), convective airflow drying (CAD), and swell drying (SD).

##### Freeze Drying (FD)

Frozen chokecherries (100 g) were freeze dried over 24 h of processing divided into two stages: sublimation (−20 °C, 6.3 Pa/12 h) and desorption (25 °C, 6.3 Pa/12 h). Freeze drying was carried out in a LabConco FreeZone Triad equipment (Kansas City, MO, USA), and obtained samples were recorded as FD.

##### Convective Airflow Drying (CAD)

Frozen chokecherries (100 g) were dried in a hot air dryer (Polinox, Ciudad de Mexico, Mexico) with air at 50 °C, 265 Pa partial pressure of vapor, and 1.2 m/s of air flux. Drying was carried out over 24 h until 11.47% humidity (wb) was obtained, and obtained samples were recorded as CAD.

##### Swell Drying (SD)

**Figure 8:**
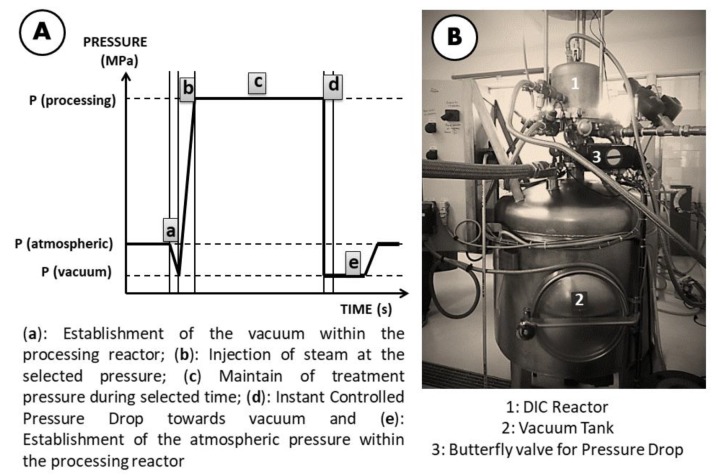
(**A**) Schematic time-pressure profiles of the instant controlled pressure drop (DIC) processing cycle and (**B**) DIC reactor LABIC0.1. (ABCAR DIC Process).

Swell drying is a DIC-assisted classical hot air drying. In the case of chokecherries, it consisted of three processing stages: (1) predrying, (2) DIC treatment, and (3) postdrying. The predrying stage was carried out under the same conditions as that of CAD, till 20% w.b. was reached. Once the predrying step finished, chokecherries were treated by DIC. Predried samples were placed inside the DIC reactor, and an initial vacuum of 4.5 kPa was established. After that, saturated steam was injected into the reactor (0.24–0.46 MPa), and it was maintained for a given time (10–22 s). When the treatment time was recorded, an instant controlled pressure drop was carried towards a vacuum (4.5 kPa). After the vacuum stage period, the pressure was released toward the atmospheric pressure, and chokecherries samples were removed from the reactor. DIC treatment was carried out on a DIC laboratory equipment (LABIC0.1, ABCAR DIC Process, La Rochelle, France). Figure 8 shows the schematic time-pressure profiles of a DIC processing cycle and the DIC reactor.

To evaluate the effect of DIC operating parameters, a central composite rotatable design was employed. Steam pressure (P) and thermal treatment time (t) were the independent variables. The design yielded 13 experiments with four (2^2^) factorial points, four star points (−α, −1, 0, +1, and +α) and five central points (0,0). The experimental design was run in random order to minimize the effects of unexpected variability on observed responses due to external factors. In each run, 100 g of chokecherry flesh were used. Table 4 shows the experimental design for chokecherries DIC processing.

After DIC treatment, chokecherries samples were postdried during 4 h under the same conditions as CAD. Obtained samples were labeled as SD.

All dried samples (CAD, FD, and SD) were packed in airtight bags and stored at room temperature until further assessments.

#### 4.2.2. Proximal Analysis

Moisture, ash, protein, fat, and crude fiber were analyzed according to the AOAC Official Methods 930.15, 942.05, 981.10, 920.39 and 962.09, respectively [28].

#### 4.2.3. Bioactive Compounds Quantification

To evaluate the effect of the studied freezing and drying processes on the antioxidant content and the antioxidant activity of chokecherry fruit, the total phenolics content (TPC), the total flavonoids content (TFC), the kuromanin concentration (KC), and the antioxidant activity (antiradical activity (ARA) and the Trolox equivalent antioxidant capacity assay (TEAC) were measured.

##### Methanolic Extracts Preparation

Prior to extraction, chokecherries samples were milled and sieved through 60 mesh. Subsequently, 0.5 g of chokecherries were extracted with 10 mL of methanol with 1% of HCl. The extraction was carried out under agitation for two hours, at room temperature (25 °C), in absence of light. To obtain the methanolic extract, sample suspensions were centrifuged (Hermle Z 383 K, Wehingen, Germany) at 6000 rpm for 10 min at 4 °C. For their further analysis, the supernatants were stored at −20 °C for less than a week. Extracts were prepared by duplicate.

##### Total Phenolics Content

Total phenolic content was estimated by using the Folin–Ciocalteu colorimetric method [29]. Briefly, 0.02 mL of the extract was oxidized with 0.1 mL of 0.5 N Folin–Ciocalteu reagent; after 5–8 min, the reaction was neutralized with 0.3 mL sodium carbonate solution (20%). The absorbance values were obtained by the resulting blue color at 760 nm with a Spectrophotometer (xMark Microplate Spectrophotometer, BioRad, Osaka, Japan) after incubation for 2 h at 25 °C. Quantification was done based on a standard curve of gallic acid ranging from 0 to 500 μg/mL. Results were expressed as mg of gallic acid equivalent per 100 g of dry matter (mg gallic acid eq./100 g d.b.).

##### Total Flavonoids Content

Flavonoid content was determined according to Oomah, et al. [30]. Briefly, the method consisted of mixing 50 μL of the methanolic extract with 180 μL of distilled water and 20 μL of a solution of 10 g/L 2-aminoethyldiphenylborate in a 96-well microtitration flat-bottom plate. The absorbance of the solution was measured at 404 nm after 15 min of reaction, with a spectrophotometer (xMark Microplate Spectrophotometer, BioRad). Extract absorption was compared with a rutin standard (0 to 200 μg/mL). Flavonoids content was expressed as mg rutin equivalent per 100 g of dry matter (mg eq. rutin/100 g db).

##### Anthocyanins Content by HPLC

Chokecherry extracts were filtered through a 0.45 μm nylon membrane filter. HPLC analysis was performed using an Agilent 1200 HPLC System (Agilent Technology 1200 series, Palo Alto, CA, USA), equipped with a quaternary pump, autosampler, and a diode array detector. Separation of anthocyanins was performed using an Agilent Eclipse XDB-C18 column (5 μm, 4.6 mm, 150 mm) at 28 °C. The mobile phase was constituted of 4.0% phosphoric acid (A) and 6.0% pure methanol (B) at a flow rate of 1 mL/min. The linear gradient conditions were 0 min 6% B, 15 min 30%, 18 to 20 min 60% B, 21 to 22 min 100% B, 23 to 25 min 6% B. Cyanidin 3-O-β-glucopyranoside (kuromanin) was used as standard (0 to 1 mg/mL). The anthocyanin concentration was expressed as µg kuromanin equivalent per 100 g of dry matter (µg kuromanin eq./100 g of d.b.).

##### Antiradical Activity by DPPH (ARA)

Measurement of antiradical activity (ARA) was carried out using 2,2-diphenyl-1-picrylhydrazyl (DPPH) as a free radical. The reduction of DPPH by an antioxidant or by a radical species results in an absorbance decrease at 520 nm. Then, 20 μL of the extract was mixed with 200 μL DPPH (125 μM in 80% methanol). After 90 min, the plate was read at 520 nm in a spectrophotometer (xMark Microplate Spectrophotometer, BioRad) and the antioxidant capacity was calculated as a percentage of DPPH discoloration (ARA%) according to Burda and Oleszek [31] and Fukumoto and Mazza [32]. The control was a mixture of 200 μL 125 μM DPPH + 20 μL of methanol. The analysis was performed in triplicate.

##### Trolox Equivalent Antioxidant Capacity (TEAC)

The Trolox equivalent antioxidant capacity assay (TEAC) is based on the ability of the antioxidant to scavenge the blue-green colored ABTS radical cation. This assay is applicable to both lipophilic and hydrophilic antioxidants. The TEAC assay was performed according to Re, et al. [33]. The ABTS radical cation (ABTS•+) was produced by mixing ABTS (7 mM) with potassium persulphate (2.45 mM). The reaction mixture was stored at room temperature in the dark for 12 to 16 h. The ABTS•+ stock solution was diluted with ethanol to an absorbance of 0.8 ± 0.1 at 734 nm. Next, 0.02 mL of Trolox standard (from 0 to 500 μM) or sample and 0.2 mL of reagent were added to a well in a 96-microplate and mixed thoroughly. The absorbance readings were taken at 734 nm after 6 min using a visible-UV microplate reader (xMark Microplate Spectrophotometer, BioRad). The TEAC of the samples was calculated as the μM of Trolox needed to give the same degree of discoloration than samples (µM eq. of Trolox). Each sample was analyzed in triplicate.

#### 4.2.4. Statistical Analysis

Statistical analysis was performed using the Statistica Software 2017 (TIBCO Software Inc., Palo Alto, CA, USA). For each treatment analysis of variance (ANOVA) (*p* < 0.05) and multiple comparisons by Tukey’s honest significant test (α < 0.05) was applied to evaluate any significant difference. In the case of the experimental design of DIC treatment, statistical analysis was also performed by the surface response methodology and the Pareto chart. The Pareto chart was used to identify the impact of variables on the responses. The vertical line in the Pareto chart determines the effects that are statistically significant at the 95% confidence level. Surface response methodology plots were used to optimize the responses. Saturated steam pressure (MPa) and thermal treatment time (s) were studied as independent variables. The studied response variables were: total phenolics content (TPC), total flavonoids content (TFC), kuromanin concentration (KC), and antioxidant activity (ARA and TEAC).

## 5. Conclusions

This study compared the impact of the following four food processing methods: convective airflow drying (CAD), freezing (FR), freeze drying (FD), and swell drying (SD) on the bioactive molecules of chokecherry fruit (*Prunus virginiana* L.), including total phenolics content (TPC), total flavonoids content (TFC), kuromanin concentration (KC), and antioxidant activity (ARA and TEAC).

The obtained results showed that both freezing and swell drying effectively preserve the TPC, TFC, KC, and ARA. Specifically, in the case of swell drying, the highest concentration of TFC and KC was found after the SD9 treatment (P = 0.27 MPa and t = 20 s), for TPC and ARA, the best results were found after the SD1 treatment (P = 0.35 MPa and t = 16 s). Moreover, the SD1 samples also presented the best antioxidant activity by TEAC. This means that with proper adjustments of the DIC operating parameters, it is possible to preserve the antioxidant content and activity of chokecherries at ambient temperature. Contrary, in the case of CAD, it was observed that this method caused the highest losses of both the antioxidant content and the antioxidant activity of chokecherries. These results could be linked to long thermal treatments, shrinkage, and collapse of chokecherries structure.

Swell drying is a key tool to remedy the shrinkage and collapsing of dried food structure, which results in better antioxidants extraction and better whole quality in terms of food safety and organoleptic aspects. Moreover, DIC can be easily adopted at an industrial scale.

Finally, although, in this study, all drying processes were applied after a previous freezing stage of chokecherry fruits, further studies are needed to evaluate the effect of CAD and SD directly on fresh fruits. Moreover, it would be interesting to perform drying and phytochemical extraction kinetics to optimize both processes. In addition, for a better understanding of the impact of the different freezing and drying studied methods, it would be interesting to study the microstructure of chokecherry samples through an electronic scanning microscope (SEM) analysis.

## Figures and Tables

**Table 1:** Water content, total phenolics content (TPC), and total flavonoids content (TFC) of frozen and dried chokecherries.

Treatment	Water Content ^1^	TPC ^2^	TFC ^3^
FR	83.34 ± 2.09	72.81 ± 4.52 ^a^	7.02 ± 0.40 ^b,c,d^
FD	14.83 ± 0.51	51.32 ± 2.65 ^f,g^	7.19 ± 0.34 ^b,c^
CAD	11.47 ± 2.47	50.29 ± 4.19 ^f,g^	6.10 ± 0.63 ^f,g^
SD1	12.95 ± 0.20	65.57 ± 5.46 ^b^	6.65 ± 0.33 ^d,e,f^
SD2	12.04 ± 0.81	52.29 ± 2.44 ^e,f^	6.66 ± 0.30 ^d,e,f^
SD3	13.43 ± 2.10	57.80 ± 4.07 ^d,e^	7.16 ± 0.18 ^b,c^
SD4	13.79 ± 0.20	63.43 ± 6.53 ^b,c^	6.86 ± 0.24 ^c,d,e^
SD5	12.44 ± 0.88	45.63 ± 3.75 ^g,h^	7.26 ± 0.32 ^b^
SD6	12.38 ± 0.19	52.30 ± 3.82 ^e,f^	6.68 ± 0.17 ^d,e^
SD7	13.07 ± 1.69	53.54 ± 3.02 ^d,e,f^	6.90 ± 0.23 ^b,c,d^
SD8	12.46 ± 2.14	57.37 ± 3.51 ^d,e^	6.87 ± 0.64 ^c,d,e^
SD9	12.12 ± 1.63	43.53 ± 3.13 ^h^	7.81 ± 0.32 ^a^
SD10	14.20 ± 0.33	58.33 ± 11.70 ^c,d^	6.81 ± 0.06 ^d,e^
SD11	12.47 ± 2.07	48.41 ± 4.23 ^g^	5.88 ± 0.31 ^h^
SD12	10.35 ± 2.47	55.41 ± 4.88 ^d,e,f^	6.29 ± 0.21 ^f,g^
SD13	13.81 ± 0.86	53.85 ± 2.16 ^d,e,f^	6.60 ± 0.22 ^e,f^

FR, freezing; FD, freeze drying; CAD, convective airflow drying; and SD, swell drying. ^1^ Water content percentage (wet basis); ^2^ TPC = mg Gallic acid eq./100 g d.b. and ^3^ TFC = mg Rutin eq./100 g d.b. Values are expressed as means of two independent experiments in triplicate (n = 6). Letters in the same column indicate significative differences Tukey HSD, *p* < 0.05.

**Table 2:** Kuromanin concentration (KC) of frozen and dried chokecherries.

	Peak 1	Peak 2	Peak 3	Peak 4	Peak 5	Peak 6	KC
FR	66.04 ± 3.02				30.53 ± 1.35	39.92 ± 1.02	758.06 ± 21.39 ^a^
FD	375.35 ± 3.66	250.84 ± 2.58	115.85 ± 5.42	29.62 ± 2.64	517.82 ± 5.24	494.47 ± 9.76	640.24 ± 35.99 ^b^
CAD	204.76 ± 2.11	153.09 ± 76.4	119.68 ± 26.80	53.32 ± 8.81	492.64 ± 34.80	682.42 ± 105	107.99 ± 1.61 ^j^
SD1	103.25 ± 5.97	10.10 ± 1.16	11.77 ± 1.69		55.01 ± 2.32	60.85 ± 2.55	485.31 ± 112.21 ^c,d,e^
SD2	85.52 ± 1.38				31.92 ± 0.23	37.59 ± 2.16	350.26 ± 90.91
SD3	120.22 ± 4.92	8.41 ± 0.52	10.96 ± 0.17		51.24 ± 1.25	51.92 ± 3.40	472.01 ± 82.48 ^d,e,f^
SD4	102.45 ± 3.07	5.80 ± 1.28	8.21 ± 0.57		45.55 ± 2.24	46.61 ± 3.92	451.96 ± 80.18 ^d,e,f^
SD5	80.91 ± 5.35				27.97 ± 0.99	30.04 ± 2.36	305.51 ± 65.89 ^h,i^
SD6	71.82 ± 1.50	17.03 ± 1.73				10.25 ± 7.26	260.69 ± 53.70 ^i^
SD7	98.46 ± 4.07	4.68 ± 1.10	7.16 ± 0.74		44.02 ± 1.83	45.98 ± 1.82	364.55 ± 42.29 ^g,h^
SD8	109.28 ± 5.11	8.93 ± 0.60	11.14 ± 1.07		53.86 ± 1.39	58.75 ± 1.48	515.83 ± 75.24 ^c,d^
SD9	99.48 ± 4.38	6.77 ± 1.38	8.97 ± 1.94		47.89 ± 3.32	54.55 ± 4.00	558.39 ± 29.59 ^c^
SD10	104.95 ± 7.72	7.84 ± 0.74	11.69 ± 0.36		51.80 ± 1.67	58.67 ± 2.51	406.91 ± 50.94 ^f,g^
SD11	98.65 ± 4.43	8.43 ± 0.65	10.75 ± 0.59		52.30 ± 1.13	60.80 ± 3.89	404.13 ± 95.59 ^f,g^
SD12	81.96 ± 5.53	1.18 ± 0.84	5.35 ± 0.89		38.80 ± 1.94	45.91 ± 1.95	421.65 ± 59.58 ^e,f,g^
SD13	86.17 ± 5.33	2.79 ± 0.29	7.69 ± 1.13		42.51 ± 1.73	50.85 ± 4.32	417.52 65.52 ^e,f,g^

FR, freezing; FD, freeze drying; CAD, convective airflow drying; and SD, swell drying. Anthocyanins content = µg kuromanin eq./100 g of d.b. Values are expressed as means of two independent experiments in triplicate (n = 6). Letters in the same column indicate significative differences Tukey HSD, *p* < 0.05.

**Table 3:** Antioxidant capacity of treated chokecherries.

Treatment	ARA (% of DPPH Discoloration)	TEAC (µM eq. of Trolox)
FR	61.38 ± 0.46 ^a^	436.26 ± 8.34 ^c,d^
FD	49.18 ± 1.57 ^d^	450.55 ± 12.40 ^b,c^
CAD	33.97 ± 0.77 ^g^	111.30 ± 22.74 ^h^
SD 1	62.89 ± 2.88 ^a^	472.68 ± 5.01 ^a^
SD 2	52.80 ± 1.35 ^c^	427.66 ± 5.36 ^d^
SD 3	56.24 ± 3.56 ^b^	430.57 ± 4.44 ^d^
SD 4	54.34 ± 6.09 ^b,c^	410.47 ± 14.52 ^e^
SD 5	48.65 ± 1.68 ^d^	340.17 ± 21.90 ^g^
SD 6	51.96 ± 0.85 ^c^	362.80 ± 3.82 ^f^
SD 7	52.93 ± 1.31 ^c^	461.30 ± 19.90 ^a,b^
SD 8	48.62 ± 0.45 ^d^	421.22 ± 2.93 ^d,e^
SD 9	40.64 ± 2.01 ^f^	376.08 ± 17.82 ^f^
SD 10	42.77 ± 1.38 ^e,f^	366.84 ± 7.03 ^f^
SD 11	42.68 ± 3.77 ^e,f^	326.00 ± 22.86 ^g^
SD 12	45.22 ± 0.89 ^e^	373.93 ± 19.90 ^f^
SD 13	41.89 ± 0.73 ^f^	325.25 ± 9.99 ^g^

FR, freezing; FD, freeze drying; CAD, convective airflow drying; and SD, swell drying. Values are expressed as means of two independent experiments in triplicate (n = 6). Letters in the same column indicate significative differences, Tukey HSD, *p* < 0.05.

**Table 4:** Experimental design.

DIC													
Run	1	2	3	4	5	6	7	8	9	10	11	12	13
Pressure, P (MPa)	0.35	0.46	0.35	0.35	0.43	0.43	0.35	0.27	0.27	0.35	0.24	0.35	0.35
Time, t (s)	16	16	22	16	20	12	16	12	20	16	16	10	16
